# The faculty-to-faculty mentorship experience: a survey on challenges and recommendations for improvements

**DOI:** 10.1098/rspb.2023.0983

**Published:** 2023-12-13

**Authors:** Sarvenaz Sarabipour, Natalie M. Niemi, Steven J. Burgess, Christopher T. Smith, Alexandre W. Bisson Filho, Ahmed Ibrahim, Kelly Clark

**Affiliations:** ^1^ Institute for Computational Medicine and Department of Biomedical Engineering, Johns Hopkins University, Baltimore, MD, USA; ^2^ Center for Teaching Excellence and Innovation, Johns Hopkins University, Baltimore, MD, USA; ^3^ Department of Biochemistry and Molecular Biophysics, Washington University School of Medicine, Saint Louis, MO, USA; ^4^ Carl R. Woese Institute for Genomic Biology and Department of Plant Biology, University of Illinois at Urbana-Champaign, Urbana, IL, USA; ^5^ Office of Research and Innovation, Virginia Polytechnic Institute and State University, Blacksburg, VA, USA; ^6^ Department of Biology, Brandeis University, Waltham, MA, USA

**Keywords:** mentoring, faculty development, research culture, early careers, peer mentoring, mentorship programmes

## Abstract

Faculty at research institutions play a central role in advancing knowledge and careers, as well as promoting the well-being of students and colleagues in research environments. Mentorship from experienced peers has been touted as critical for enabling these myriad roles to allow faculty development, career progression, and satisfaction. However, there is little information available on who supports faculty and best ways to structure a faculty mentorship programme for early- and mid-career academics. In the interest of advocating for increased and enhanced faculty mentoring and mentoring programmes, we surveyed faculty around the world to gather data on whether and how they receive mentoring. We received responses from 457 early- and mid-career faculty and found that a substantial portion of respondents either reported having no mentor or a lack of a formal mentoring scheme. Qualitative responses on the quality of mentorship revealed that the most common complaints regarding mentorship included lack of mentor availability, unsatisfactory commitment to mentorship, and non-specific or non-actionable advice. On these suggestions, we identify a need for training for faculty mentors as well as strategies for individual mentors, departments, and institutions for funding and design of more intentional and supportive mentorship programmes for early- and mid-career faculty.

## 1. Introduction

Mentorship plays an important role in academic success by aiding researchers' well-being and career development. For academic faculty, mentorship can lead to tangible benefits including higher career satisfaction [1], increased sense of self-efficacy [2,3], an expanded professional network [4], greater likelihood in obtaining funding [5], an increased number of publications [6,7], more time spent on research [8], a shorter period to tenure [9] and improved retention in academia [10]. Additionally, mentorship can assist early career faculty in adjusting to new and demanding expectations in roles for which they have little training such as balancing research, mentorship and teaching loads, managing budgets, and navigating departmental politics. As few postdoctoral researchers receive comprehensive training in all of these areas, mentors can assist in easing the transition into the faculty role through their advising, experience, and wisdom. Despite these benefits, access to and quality of mentorship may not be tailored to mentee needs, leading to variable mentorship experiences [11]. Such variability can limit potential mentorship benefits, and increasing access to effective mentors could improve the well-being and overall performance of faculty.

Researchers in the science of mentorship have identified determinants of positive mentoring relationships [11,12], producing some recommendations on how to implement these at the institutional level [13,14]. This led to the realization that mentoring can be taught and aided by the development of curricula [15], tools for practice, and assessment [16,17]. However, many faculty may be unaware of the advantages of mentorship or lack access to pools of able or willing mentors—issues that may be more pronounced for women or marginalized communities [18,19]. Furthermore, faculty may lack an appreciation of the many avenues from which mentorship can be sourced. While mentoring has historically been viewed as a dyad between an experienced and a less experienced individual, it can exist in a variety of forms such as networks, peer-groups [20], group mentoring [21] or distance mentoring [22]. Mentoring can also occur on a formal (i.e., within the context of an official mentorship scheme) or informal (i.e., an independently sourced advisor not part of an official mentorship scheme) basis [14]. A lack of mentorship at any of these levels can have a negative impact on the success and retention of faculty members in academia [23]. To address this, a number of institutions have established and openly documented formal faculty programmes to increase access to mentoring [18,23–28].

Understanding mentoring needs for diverse individuals requires an understanding of the status quo to focus efforts. Systematic reviews and longitudinal studies have tracked the success of faculty mentoring programmes, but most studies are limited to individual disciplines, institutes or regions, with the majority of faculty mentoring programmes to date located in the United States [14,29]. As the benefits of mentoring are believed to be universal, studies that compare the differences in needs of faculty mentees and action of mentors, departments and institutions would be highly valuable. Unfortunately, such longitudinal studies are still sparse and disconnected. While academics commonly discuss mentorship initiatives for trainees, it is not always clear who supports faculty, and, in particular, how junior faculty are mentored early in their career. To obtain an overview of mentoring experiences in research environments, we conducted a survey of early and mid-career faculty, mostly from the science, technology, engineering, mathematics and medicine (STEMM) disciplines, to gauge mentee perceptions of mentor effectiveness. We collected responses from individual faculty across six continents, implementing quantitative and qualitative questions and receiving responses on a broad range of mentoring characteristics. The results reveal that faculty mentoring experiences are not homogeneous and that there remains a need for implementing constructive mentorship initiatives and relationships that are tailored to individual faculty members.

## 2. Methods

### (a) Survey participant recruitment

The text for the survey used in this work is included in the electronic supplementary material. A Google Form was used to conduct the survey and was distributed on social media platforms via academic Slack groups (i.e., the New PI Slack and the Mid-Career PI Slack) and via group leaders/faculty/principal investigators on X (formerly Twitter) worldwide. The survey was distributed from March 2019 to March 2020 and contained both scaled-response and open-ended questions. Responses to the first survey question with ‘0’ for number of mentors, were routed out of the data analysis for certain analysis as specified in the electronic supplementary material, tables supporting the figures. The respondents to the survey were asked to self-report, and the information collected was not independently verified.

### (b) Data analysis

**Figure 1.**
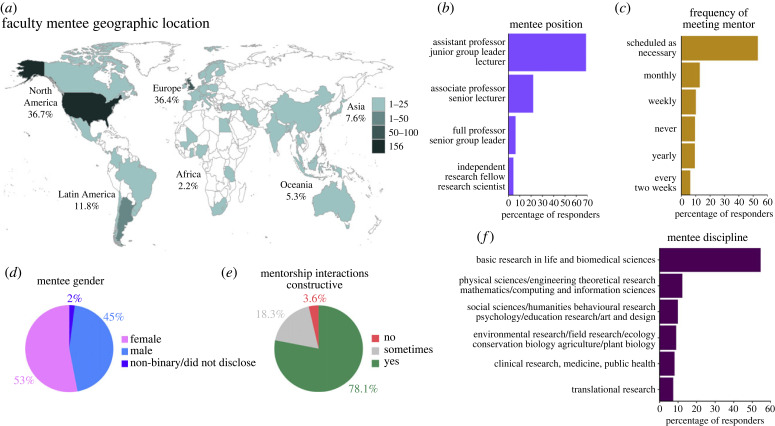
Mentee demographics. Distribution of survey respondents by (*a*) country of research (institutional affiliation) for the 457 early- and mid-career faculty; (*b*) mentee academic positions included assistant professors, lecturers (a term commonly used in United Kingdom), junior group leaders (a term mostly used in Europe) and associate professors (with or without tenure); (*c*) mentee–mentor meeting frequency; (*d*) mentee gender distribution; (*e*) quality of mentee–mentor interactions; (*f*) mentee scientific discipline. All responses were self-identified (electronic supplementary material, tables S1, S3, S4, S6, S7, S9).

**Figure 2.**
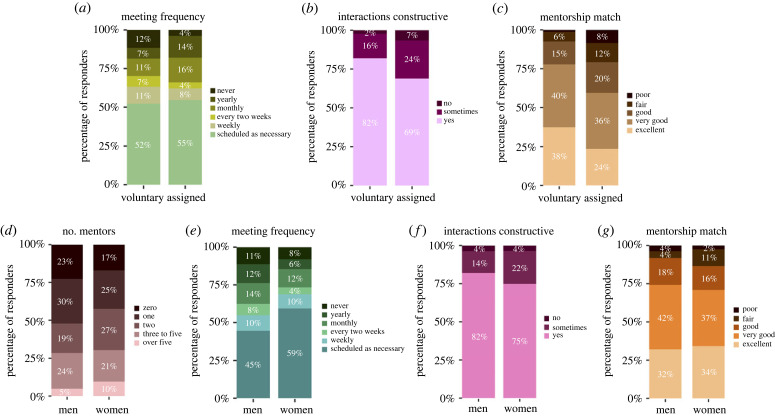
Mentorship features and quality by gender and mentorship initiation mode (faculty mentee chose mentor voluntarily or mentor was assigned to faculty mentee by their department). (*a,e*) Mentee-mentors meeting frequency. (*b,f*) Constructiveness of mentorship interactions. (*d*) Number of mentors. (*c,g*) Quality of mentorship match. Data analysis for (*a*), (*b*), (*c*), (*e*), (*f*), (*g*) excludes survey respondents with ‘0’ number of mentors (electronic supplementary material, tables S9, S10, S11, S14, S9a, S10a, S11a, S14a, statistical analysis, figure S10, table S41).

**Figure 3.**
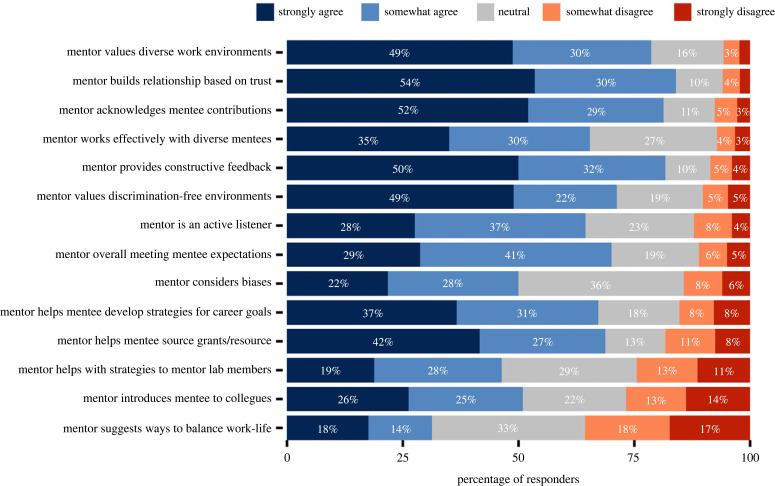
Characterization of mentor–mentee interactions with respondents evaluating their mentorship on a Likert scale (electronic supplementary material, tables S23–S36).

**Figure 4.**
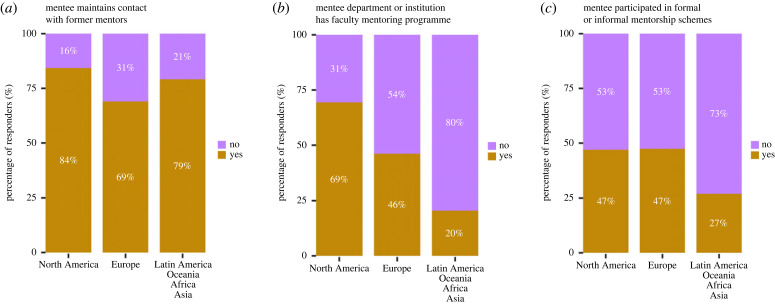
Mentorship characteristics by geographical location. (*a*) Respondents maintaining contact with former mentor(s). (*b*) Existence of faculty mentoring programme to mentor the faculty mentee on their own career. (*c*) Respondents participated in any formal (i.e., run by institutions) or informal programmes (electronic supplementary material, tables S15a, S18a, S19a, statistical analysis, figure S12, table S43).

Microsoft Excel, RStudio, ggplot package and the eulerr package were used to graph the results shown in figures 1–4, electronic supplementary material, figures S1–S8. The qualitative survey comments were categorized by theme (keywords, themes and context) describing each comment and the frequency of comments pertaining to a particular theme are summarized in Results and Discussion. Bar plots were generated from distinct themes in qualitative responses (electronic supplementary material, figures S7–S8). For statistical analyses, Prism 9 was used to perform ordinary one-way analysis of variance (ANOVA) (electronic supplementary material, figures S9, S14), two-tailed Mann–Whitney *U* (Wilcoxon rank sum) test (electronic supplementary material, figures S10, S11) and the Tukey multiple comparisons test (electronic supplementary material, figures S12, S13, S14).

## 3. Results

### (a) Characteristics of faculty mentee demographics and mentorship interactions

To understand how faculty perceive their professional mentorship, we surveyed group leaders worldwide to assess mentoring practices and efficacy. As terminology differs across countries, ‘faculty’ and ‘group leader’ are used interchangeably within this study and include lecturers and faculty at the assistant or associate professor level, with or without tenure. The survey was distributed from March 2019 to March 2020 and contained both scaled-response and open-ended questions. The survey asked respondents at senior career stages (e.g., full professors) to participate with regards to mentorship they received at early and mid-career stages. These responses constitute, to our knowledge, the largest and broadest dataset on faculty mentoring practices and their effectiveness worldwide. We received responses from 457 faculty from 48 countries (figure 1*a*). Almost one-third of our responses were from faculty working in the United States (35%) followed by the United Kingdom (16%), Argentina (8%), Australia (5%), Spain (5%) and 1–3% of our responses from 43 other countries each. Sixty-eight per cent of respondents were assistant professors (or equivalent rank) while 22% were associate professors (or equivalent rank), 6% were full professors and 4% identified as independent research fellows (figure 1*b*). Most respondents (53%) met with mentors on an as-needed basis (figure 1*c*). Ninety-five per cent of respondents worked at an academic institution (electronic supplementary material, figure S1a). Respondents were relatively evenly distributed across self-identified genders, with 45% identifying as male, 53% as female, and 2% identified as non-binary or preferred to not to disclose this information (figure 1*d*). Overall, 78% of respondents found their interactions with their mentor constructive (figure 1*e*). Notably, the majority (90%) of respondents performed research in STEMM disciplines. Fifty-four per cent of respondents performed basic research in life and biomedical sciences, 12% in physical and mathematical sciences, computer science or engineering, 10% in social and behavioural sciences and humanities, 9% in environmental sciences and field research, 8% in clinical and medical research and 7% in translational research (figure 1*f*) and 91% held a PhD (electronic supplementary material, figure S1b).

To understand how faculty mentorship is practised across institutions and disciplines, we queried if mentorship was taking place and, if so, how mentoring relationships typically functioned among our respondents. Faculty use a combination of formats to receive mentorship and meet with mentors (electronic supplementary material, figure S1c). Most faculty mentees chose their faculty mentors voluntarily (70%) while a minority had a mentor assigned by their department (30%) (electronic supplementary material, figure S2a). While most respondents indicated that they were receiving mentorship, a considerable fraction (approx. 20%) of respondents reported not having a mentor, 27% had one mentor, and 53% had two or more mentors (electronic supplementary material, figure S2b). Over 80% of respondents worked with their mentor for at least 1 year (electronic supplementary material, figure S2c) and 64% were in the 35–45 age group (electronic supplementary material, figure S2d). Only 11% of mentees described the quality of match with their mentor as poor-to-fair, while 89% regarded their mentoring experience as good-to-excellent (electronic supplementary material, figure S2e).

### (b) Influence of mentorship initiation mode and gender on mentorship satisfaction

Analysis of responses by mentorship initiation format showed that faculty mentees who had an assigned mentor met with similar frequency with their mentor compared to faculty who voluntarily chose their mentor (figure 2*a*, electronic supplementary material, S10a, *p* = n.s.). Faculty mentees who had an assigned mentor found their mentorship interactions less constructive (figure 2*b*, electronic supplementary material, S10b, *p* < 0.01), and were less satisfied with the mentorship match (figure 2*c*, electronic supplementary material, S10c, *p* < 0.001), which remained consistent across genders (electronic supplementary material, figure S9b) compared to those who chose their mentor voluntarily. We assessed the survey results for possible trends on gender disparities in access to and satisfaction with mentors. 23% of men and 17% of women lacked mentors, with no significant difference between genders (figure 2*d*, electronic supplementary material, S10d, *p* = n.s.) and 58% of women having more than one mentor compared to 48% of men. A total of 11% of men reported few to no meetings with their mentor as compared to 8% of women, with no significant difference between genders (figure 2*e*, electronic supplementary material, S10e, *p* = 0.0593, *p* = n.s.). Twenty-six per cent of women and 18% of men reported their mentorship interactions to be ‘not constructive’ or ‘sometimes constructive’, with no significant difference between genders (figure 2*f*, electronic supplementary material, S10f, *p* = n.s.). Men and women had similar neutral to negative and fair-to-poor mentorship match (figure 2*g*, electronic supplementary material, S10g, *p* = n.s.). Twenty-four per cent of women than men and 17% of men did not maintain contact with former mentors (electronic supplementary material, figure S6). The majority of mentees of both genders reported having a male mentor (71% of men and 60% of women) (electronic supplementary material, figure S6) consistent with persistent faculty gender gap across disciplines [30–33].

Half of respondents did not have a faculty mentoring programme in their department or institution, and most reported that they had not participated in such a programme (52% of women and 65% of men) (electronic supplementary material, figure S6). Despite this, 75% of women and 76% of men reported having at least one mentor within their department or institution, while 25% reported having additional mentors at other institutions (electronic supplementary material, figure S6).

We asked respondents to rate the characteristics of their mentor and the extent to which their mentor met their expectations. This included questions on how their mentor interacted with them (e.g., used active listening, was trustworthy, provided constructive feedback), whether their mentor advocated for them (e.g., acknowledged professional contributions, helped in acquiring important resources, and helped strategize and prioritize goals), whether the mentor ensured a hospitable working environment (e.g., decreased or eliminated workplace discrimination and harassment, valued and promoted diverse backgrounds, and acknowledged potential biases and prejudices). The most pressing issues that mentors could improve upon were advising on work–life balance (negatively perceived by 36% of women and 29% men), networking and introducing mentee to colleagues (negatively perceived by 33% of women and 18% of men), guidance on mentoring laboratory members (negatively perceived by 29% of women and 20% of men), sourcing grants or other resources (negatively perceived by 18% of women and 18% of men), and strategizing mentee career goals (negatively perceived by 17% of women and 13% of men) (figure 3, S15). However, these issues were perceived similarly across genders, with no significant differences across these comparisons (electronic supplementary material, figure S11).

### (c) Faculty mentorship across continents

In analysing responses on mentorship, trends suggest differences in number of mentors, and mentee valuation of mentorship interactions based on mentee geographical location. We compared North America, Europe, and ‘all other continents combined’ as this resulted in reasonably sized subdivisions of our data. Faculty in Europe and other continents had fewer mentors (in Europe 27% had zero mentors, 73% had one or more mentors) compared to faculty in North America (11% had zero mentors, 89% had one or more mentors) (electronic supplementary material, figure S3a, S13a, *p* < 0.001). Further, fewer European PIs (39%) had multiple mentors compared to North American PIs (75%). Respondents across continents met with similar frequency (weekly, every two weeks, monthly or yearly) with their mentors (32%) compared to North American faculty (36%) (electronic supplementary material, figure S3b, S13b, *p* = n.s.). Respondents across continents found their mentorship interactions constructive to similar levels (electronic supplementary material, figure S3c, S13c, *p* = n.s.). Both European and North American faculty expressed similar good-to-excellent mentorship matches (87% versus 92%) (figure 3*d*, electronic supplementary material, S13d, *p* = n.s.). Interestingly, there was no significant difference in mentorship quality reported for mentees with more mentors across geographical regions (electronic supplementary material, figure S9c, *p* = n.s.). These data reveal that North American faculty report significantly more mentors than faculty in other continents (electronic supplementary material, figure S9d), but this does not translate into significant differences in mentorship quality (electronic supplementary material, figure S9e). Thirty-one per cent of European group leaders did not maintain contact with former mentors compared to 16% of North American faculty with only significant differences between North America and European faculty mentees (figure 4*a*, electronic supplementary material, S12a, *p* < 0.001). Significantly more departmental or institutional faculty mentoring programmes were reported to have been provided to North American group leaders relative to faculty in Europe or other continents (figure 4*b*, electronic supplementary material, S12b, *p* < 0.001 and *p* < 0.0001). European and North American faculty were also significantly more likely to participate in peer mentorship programmes than faculty in other continents combined (figure 4*c*, electronic supplementary material, S12c, *p* < 0.01). Only 47% of European and North American faculty and 27% of faculty in other continents combined participated in a formal or informal mentorship programme.

### (d) Academic satisfaction and career optimism

The survey further inquired on faculty mentee satisfaction and optimism about their current and future research and position. The data show that women were less satisfied with their research progress (32% of women versus 19% of men) and less optimistic about the future of their career (27% of women versus 14% of men) (electronic supplementary material, figure S4a,b, S5a,b, S14a,b, *p* < 0.05). Analysis of responses by mentorship initiation format showed mentees who had assigned mentors and mentees who had chosen their mentor voluntarily were similarly satisfied with their research progress (electronic supplementary material, figure S4c,d, S5c,d, S14c,d, *p* = n.s.). Analysing responses across continents, faculty in Europe and other continents expressed similar satisfaction with their research career, but significantly less satisfaction compared to North American faculty (electronic supplementary material, figure S4e, S5e, S14e, *p* < 0.05). Similarly, North American respondents and expressed higher career optimism compared to group leaders from Europe or all other continents (electronic supplementary material, figure S4f, S5f, S14f, *p* < 0.001). While presence or absence of mentors may be a significant contributing factor to respondents' academic satisfaction and career optimism, differences in funding and career stability, biases, and other structural issues are additional likely contributing factors.

### (e) Analysis of qualitative responses

We asked respondents, in the form of long response questions, if their interactions with mentors were constructive, and if not, what they were seeking in the mentorship relationship. Using these data, we summarized key features displayed by helpful mentors that were noted as helpful (electronic supplementary material, figure S7) or unhelpful (electronic supplementary material, figure S8). Many of the issues raised by mentees appear to be caused by poor alignment of expectations, including mentees' want for emotional support, professional development, career guidance, and sponsorship. Through a compilation of responses from mentees on their mentoring relationships, we have identified common pitfalls in mentorship as noted by faculty mentees.

### (f) Mentorship is of variable accessibility and quality to faculty

Survey responses indicated a wide range of access to mentorship for faculty. Concerningly, 20% of all respondents did not receive any substantive mentoring upon their transition to an independent investigator position (electronic supplementary material, figure S2b), but the underlying causes fueling this lack of mentorship varied. Some faculty reported organizational failures in promoting mentorship due to a lack of formal mentoring programmes or an absence of mentoring culture. Some respondents noted that although they were assigned an individual mentor, this mentor failed to understand what type of support the mentee needed, leaving the mentee unsupported. Our data indicate that most faculty desire mentorship from more experienced faculty, and suggest that in many contexts, current mentorship plans and programmes could be improved to minimally enable all early and mid-career faculty access to at least one experienced mentor.

Those receiving mentorship reported a large range in the quality of their mentorship interactions. In agreement with quantitative survey responses (figure 4, electronic supplementary material, figure S3), this could be driven by geographical or cultural differences, as respondents noted that in some countries it is uncommon for faculty to source mentors at their university, whereas in others it is normal or mandatory. This variability in quality was also driven by the mechanisms in which mentoring relationships are established. Some respondents indicated that their mentor was assigned but failed to provide substantial value to their careers, leading to some respondents noting that their mentor did not appreciate or value their research. A scientific mismatch led to mentees feeling that their mentors lacked important insights on research approaches, or were unable to appreciate and vet ideas regarding their research programme. Other respondents noted that their mentor offered useful guidelines for research excellence and how to obtain research funds, but failed to ensure a working environment free of harassment and bias. Generational differences also impacted perspective and could contribute to perceived mismatches in mentorship; some respondents noted that they were unsure whether their senior mentors recognized the difficulties faced by different generations in academia. Low valuation of mentorship by senior faculty was also expressed as a common problem, which we elaborate on below.

### (g) Mentorship is not consistently valued among faculty mentors

The most common complaints regarding mentoring relationships include poor or neglected mentorship, a lack of care or availability, and disingenuous or inconsistent support. Many mentees noted having mentors who rarely met with them, resulting in an inability to foster a genuine relationship. Others reported that mentors would make time to meet only if asked repeatedly, leaving the mentee feeling as though the relationship was not valued by the mentor. However, mentees recognized that these behaviors may be driven by the inherent pressures of academia; many felt that their mentors do care about them but lack the bandwidth to engage as a mentor. This disengagement was reflected in feedback that mentors, while helpful, often did not go out of their way to provide guidance, or that when they offered direct assistance, they would fail to follow through with these offers. Still others noted that their faculty mentoring relationship was not inherently poor, but rather turned negative due to neglect or apathy over time.

Another common mentee complaint was that their mentoring relationships lack perceived value. Many noted that their mentors did not provide focused advice or career guidance and gave little to no constructive feedback to their mentees. For instance, some respondents indicated that it is common knowledge that early career faculty would need high quality papers and grants, finding this level of advice unhelpful. Responders noted that some mentors did not provide effective and constructive advice on how to achieve future goals, nor did they help to develop an actionable career plan. Some respondents noted that mentors often asserted what they would do in a situation, which sometimes was not aligned with mentee interests or made the mentee uncomfortable. Other faculty did not receive advice on how to be academically successful in either research or career development, even after soliciting such advice from their mentor.

Such failings in a mentoring relationship may stem from poor communication between mentor and mentee, which was noted by numerous survey respondents. Some stated that their contact with their mentor was through specific means (e.g., via email), restricting their interactions. Others found mentors were difficult to talk to, had poor listening skills, or projected their own experience upon mentees. However, poor communication can also originate from the mentee, as multiple respondents noted that they were not completely forthcoming about their struggles, leading to misunderstandings about their needs.

Several responses mentioned harmful and concerning mentor behaviors. Respondents noted mentors that 'forget' to include or support mentee at key moments, or those who never read material on which the mentee had requested feedback. Respondents noted mentors who provided positive feedback yet had a hierarchical attitude and belittled mentees by calling them inexperienced. Some noted poor or unrealistic advising, including recommendations that mentees abandon all projects not destined for top journals, which mentees found discouraging. A number of mentees experienced bullying, or felt that their mentor exploited their funding and junior position for their own gains by, for instance, coercing mentees to conduct experiments for their own work. Some mentors triggered conflicts between the mentee's group and their own group, causing unnecessary conflicts within their department. Other junior faculty perceived professional jealousy such that some mentors acted as if they saw mentee as a competitor for departmental resources. These behaviors are particularly concerning, as they document instances in which the mentorship of junior faculty is exploitative and would have a highly stressful and negative impact on trainees' careers.

### (h) Recommendations for improving mentorship for faculty

Based on our survey responses, we have assembled the following set of optimal characteristics desired by faculty mentees in faculty mentors, departmental leadership and institutions in faculty mentorship.

### (i) Mentors can help improve and optimize mentorship for faculty mentees

#### (i) Need for honesty, active listening, generosity, vision

One powerful outcome of our survey analysis is the emergence of a common set of qualities desired in a faculty mentor, which include honesty, trustworthiness, and an ability to have confidential discussions (electronic supplementary material, figure S7). Respondents valued mentors who made interactions with mentees relaxed, which allowed mentees the ability to speak frankly to a colleague and have transparent conversations about the highs and lows of the academic workplace. Mentees also value the opportunity to discuss topics without judgement from their mentor, and to receive unvarnished, truthful advice from an experienced perspective. Respondents desired their mentors' honest opinions about their progress and future career, and valued sincerity. Respondents particularly appreciated mentors who were not afraid to tell the mentee when something could be improved.

#### (ii) Need for supportive and knowledgeable mentors across topics

Many respondents noted that the best mentors show genuine support and concern for their careers and general well-being (electronic supplementary material, figure S7). Respondents appreciated when their mentor adapted to their unique personality and view of science, and desired mentors who offer encouragement, positive affirmation, and who hold a strong belief in mentee potential. While these qualities were consistently valued among respondents, these desires manifested in different objectives in mentoring relationships; while some respondents wanted mentors who value mentee qualities and contributions in publications (i.e., scientific contributions), other respondents noted that they value mentors on a personal level who made them feel safe and professionally valued. These differences in opinions captured in our qualitative questions highlight the various roles that mentors can have, both scientifically and personally. For example, some respondents noted being assigned a specific mentor (e.g., through a professional society) who was a fantastic mentor in professional situations, but could not offer support on other aspects of being a faculty member. This suggests that mentees would be best served by identifying multiple mentors that can each provide mentorship in distinct roles including setting up a laboratory, the navigation of academia and in managing work–life balance.

#### (iii) Need for advice setting up and running a laboratory

Mentees expressed an appreciation for mentors who offered targeted career advice when asked as well as general perspectives on common challenges. Effective mentors were reported as inquisitive, asking thought-provoking questions to both brainstorm ideas and provide focused advice. This advice was particularly useful when helping the mentee focus on their short- and long-term goals. Respondents appreciated informal advice that clarified the mentee's thinking. Respondents valued mentors who helped mentees overcome faculty career transition difficulties such selecting collaborations, hiring students, and helping mentees identify projects to build independent research programmes. Mentees noted that effective mentors neither judge nor impose their views, but rather allow the mentee to explore all possibilities before chiming in with their own opinion. These behaviors allow mentors to promote personal growth and encourage new ideas.

Respondents also wished for structured support in developing their research profile as faculty through guidance on preparing manuscripts, communicating research, hiring and promotion advice and advising on service efforts. Respondents mentioned that they appreciated mentors' advice on which conferences to attend and identifying journals to which they could submit their work. Respondents desired mentorship and advice on seeking funding, advice on how various funding agencies function, how to write grants, and how to address revisions. Some respondents noted that their mentor had been particularly helpful in reviewing their grants.

Many respondents noted that there is no real training on how to manage a laboratory and little to no support available when things go wrong. Respondents thus valued mentor advice and tips on how to recruit personnel, manage staff, and mentor their own trainees. Mentees also value advice on how to prioritize their time while minimizing energy spent on irrelevant matters (spanning both academic and work relationships). Being offered these perspectives based on experiences otherwise unavailable to mentees is a highly valued commodity among faculty. Respondents noted that the transition to independence is challenging, and that having supportive mentors substantially eases these difficulties.

#### (iv) Need for mentors to assist with navigating academia across scales

Amongst the most expressed needs of junior faculty is access to senior faculty to assist the navigation of academia. This typically spans scales, with mentees needing assistance in understanding the policies and politics of their department, their institution, and their scientific field. Respondents noted that a mentor who could speak to the necessary components for departmental success would be highly beneficial, suggesting junior faculty should seek out at least one senior member within their department from whom they can obtain this information. Ideally, this departmental mentor could also speak in an unbiased fashion about departmental politics and relevant scenarios that may impact the professional life of the mentee.

Respondents also sought useful discussion on the tenure process, mentee career progression, and establishing themselves in their scientific fields. Mentees report valuing transparent, constructive, and rigorous feedback to ensure they are on track for a successful tenure case. Respondents wished that their mentor provided appropriate support and guidance to build their career, including unbiased advice on career moves and career options or lack thereof. Mentees value mentors who anticipate areas that the mentee should consider but may not be aware of (i.e., who can flag 'unknown unknowns'). Mentees desired strategic tips regarding mid-career planning, from grant application strategies to deciding how to expand or focus the research interests of the mentee's laboratory. Thus, mentees find value in mentorship well into their established careers, as the challenges faced by faculty evolve throughout their careers.

#### (v) Need for mentors' sponsorship and advocacy

Respondents report that excellent mentors serve as their advocates and look after mentee interests. These mentors actively support career development through discussions about research and career strategy while recognizing mentee contributions to research. Among the most impactful actions that a mentor can have is to promote the mentee's career. This could include introducing the mentee to senior research leaders in their discipline or promoting mentee successes to senior institutional leadership, and to the wider community. Other respondents commented on their appreciation for mentors who tried to remove barriers to mentee progress, whether at the administrative, scientific or professional relationship level. Respondents hoped for mentors who understand their privilege and how to navigate in a changing academic culture.

#### (vi) Need for personalized mentorship for faculty

Beyond scientific and professional advice, respondents desired well-rounded mentors, noting the importance of having someone who celebrates and encourages success and growth above and beyond publishing papers and who can offer wisdom on issues such as work–life balance. Respondents noted that having mentors to advise on unique, stressful or unexpected situations, to help the mentee navigate through challenges was valuable in the transition to independence. Mentees noted that having mentors reminds them that they have a support system when they feel lost on the academic journey. This can be particularly valuable for women, underrepresented minorities, or immigrants, who would highly benefit from mentorship on specific additional challenges that each of these groups face on both a scientific and personal level. Notably, these comments highlight the heterogeneity in faculty members and thus their mentorship needs, highlighting the need for diverse mentors to help mentees on their own unique route to success.

#### (vii) Need for mentor initiative, investment and commitment

Respondents noted that mentors need to be invested in and have care for mentee success, spending time and effort to improve the mentoring relationship. Mentees particularly appreciate mentors who are generous with their time (electronic supplementary material, figure S7), noting that as a mentee it was reassuring to know that they could ask questions of their mentor without feeling that they were bothersome. Respondents noted mentors exceeding expectations with their help, at times even outside of typical work hours, and many acknowledged and appreciated their mentors taking time out of their busy schedule with no formal recognition or little benefit to them. Mentees particularly valued interacting with mentors with sufficient time to provide contextual advice, rather than standard aphorisms that apply to all. Many mentees desired a mentor who puts mentee career development and progress ahead of their personal interests and genuinely cared for the mentee's progress and success.

#### (viiii) Need for accessible and reliable mentors

Mentees highly preferred mentors who were accessible, available, and reliable. Many noted that their mentor met, when necessary (as also evident in quantitative responses figure 1*c*), and, as a result, was readily accessible to provide practical advice. An open-door policy was noted as critical, as some mentees noted that they felt as though they could only approach their mentor with important or pressing matters, or when they had specific questions to be answered. Some respondents noted that at times it was difficult to access their mentor, but they maintained a positive relationship, and when they did meet, the meetings were excellent. In these cases, the initiative to continue the mentoring relationship came almost exclusively from the mentee. Thus, the best mentors not only invest time to advise their mentees, but also take initiative in checking in on them.

Some noted the mentor being excellent and compassionate, dedicating significant time to the relationship, seeing the whole picture on how to get to the final goal (whether a grant or being published in appropriate journals), but still having a poor mentoring relationship with the mentee. This suggests that even ‘effective’ mentors can be mismatched with mentees and may lack key skills for cultivating a healthy mentoring relationship, further highlighting the need for mentor training programmes.

Some respondents noted their mentor made good suggestions, but were generally hands off, which encouraged mentee independence. This raises an interesting dichotomy, as some mentees crave a hands-off relationship to promote independence, while others perceive this as a lack of interest. Thus, open communication is critical for a solid mentorship relationship to allow dynamic interactions that can ebb and flow as the relationship progresses and as mentorship needs change. These differences in expectations also highlight the need for personalized, tailored mentorship to promote the success of junior faculty.

### (j) Departments, institutions and funders can improve mentorship for faculty

#### (i) Need for formal institutional and departmental mentors

Our survey revealed that mentors both inside and outside of a mentee's institution were important to provide guidance on the tenure process and mentee career progression. Respondents noted that formal mentorship schemes were important, as a lack of formal mentorship typically translated to few specific expectations from their mentors. In the absence of such schemes, ongoing support from a more senior colleague was deemed valuable by respondents, suggesting that informal mentorship is valued by junior faculty. Consistently, some survey respondents noted that no formal mentoring scheme exists in their institution because mentees felt they worked in a collegial environment with a low tenure bar where informal mentoring was readily available.

Respondents who only reported mentors at other institutions desired more frequent interactions with mentors, noting that at times it was difficult to get advice or feel supported. Some respondents also noted having an assigned mentor at their host institution, but that this mentor was not helpful or trustworthy. Thus, without institutional structure and active assistance from the departmental level, some faculty may not obtain necessary support to find a mentor. Respondents noted that working as a junior faculty member is difficult without a formal mentor, and, in the absence of effective mentorship, sought alternative sources for guidance. For instance, some respondents noted their postdoctoral advisor had acted as their unofficial mentor after transitioning to their faculty position, helping much more than any mentor at their current institution. Respondents also noted that having formally assigned mentors was desirable because they felt more freedom to contact their mentor, feeling less as though they were being bothersome because of the formality of the established relationship. Some also hoped that multiple formal interactions would lead to an informal mentoring relationship.

#### (ii) Need for informal mentorship

Responders noted having mentors who were not assigned to them but had mentored them of their own accord, naturally establishing informal mentoring relationships. Some responders noted that faculty mentorship should be informal and diverse, with many different faculty colleagues, including mentors in mentee's specific discipline and on an as-needed basis. Some noted that their institution had a formal mentor programme that was underwhelming, necessitating mentorship to be sourced elsewhere. Some respondents noted preferring a mentor of the same gender and outside their line management hierarchy. Others noted having a formal mentorship relationship that only made sure the mentee checked boxes for tenure. Respondents also noted that had they not sought mentors on their own, they would not have been as happy nor as effective in their job. Thus, informal mentors could serve as valuable additions to a mentorship team, each bringing different assets to mentee life and career.

#### (iii) Institutions need to offer mentorship and mentor training programmes

Some respondents noted that their university did not have formal mentoring programmes, effectively setting a low bar as to what to expect from a mentor. Mentoring programmes for early career faculty on the tenure track have recently been introduced at a number of institutions in the United States [30]. Responders to our survey noted that they would have appreciated having a mentor on-site to talk to often in person. Responders noted that not many faculty are skilled in mentoring, with training and buy-in being key for both mentors and mentees. Some respondents who had informal mentors noted that their great mentors had received training on mentoring. Some also noted that their institution did not have a mentor programme, so they felt fortunate to have a mentor, believing it should be an essential part of every academic institution. Some respondents noted that their institution was too small to have anyone who works in their specific field, suggesting that inter-institutional mentorship programmes would be valuable. Some respondents noted a complete lack of mentors for their research and for receiving tenure, resulting in their desire to change the system to be more supportive of the current junior faculty. Responders also believed that it would be impossible for academics to be expected to be good mentors without training. Therefore, provided the number of skills required to be developed to become a good mentor, each faculty mentee likely needs more than one mentor assigned with a clear expectation. This would be far more easily achieved through formal mentoring schemes, which would be enabled by institutional support for mentorship training programmes.

#### (iv) Need for multiple and diverse mentors or a mentorship team

Our survey suggests that highly effective mentorship comes from teams consisting of a diverse set of individuals and experiences as opposed to traditional mentoring dyads. Of respondents who found the mentorship they received barely acceptable, many noted that the advice they received had been valuable in only context-dependent situations, but was lacking in others. These respondents commented that their main mentors were valuable, but noted that there were some important professional aspects for which this one mentor was not well-suited, suggesting a mentorship team would be more helpful. This was true for other sets of mentees as well: some wished for a committee of mentors that were closer to their field as a strategy to mitigate the lack of knowledge or expertise from one specific mentor. Some answered the survey questions in regards to a variety of mentors (authors had asked respondents to respond with regards to their key mentor), noting that they received excellent mentoring from multiple mentors and that they had a great relationship with their primary mentor. Some responders also chose to focus on one of their closest institutional mentors in response to our survey, thinking that their shortcomings were more important to highlight. Still others noted that no one person was perfect in any specific thing and that different perspectives by multiple mentors are always illuminating, showing that there is not a single right way to attempt addressing various academic work and life challenges.

The benefits of mentoring teams were supported by comments from respondents' desired perspective from a third party who can help the mentee evaluate their standing and track record. Sometimes one has to figure out what a person is experienced at to understand who would be the best person to ask for advice on certain issues. Respondents noted having chosen a group of mentors, each for specific skills. Each mentor did a great job at their task, which was effective as the mentee did not expect one mentor to advise on all topics. Other responders noted having an equal number of men and women mentors, locally, from previous institutions and also other institutions within the geographical area. Mentees approached mentors selectively, depending on the question or problem they were facing. Other responders noted having two senior males and a senior female mentor who provide complementary information about how to advance a mentee's career. Some respondents noted having several mentors from multiple programmes or departments.

#### (v) Need for peer mentorship communities

Some respondents pointed out that as female academics, lack of mentorship and lack of academic support from senior researchers was a major contributor to the leaky pipeline and women's opportunities for the transition from postdoc to independent investigator. Some reported that other than brief interactions with mentors (2–3 people, less than 1 h per year for specific questions), that their only mentors are online resources (e.g., Twitter or online communities such as the New PI Slack or the Mid-Career PI Slack), noting that they received much more help and support to grow academically from peer mentorship on these forums. While peer support is helpful, it is not sufficient and not the same as institutional or formal mentorship from more senior faculty. This finding indicates the role of technological advances in shaping mentoring relationships, and further research is required to understand the relative benefits of social media to conventional in-person contacts.

### (k) Maintaining valuable interactions with former mentors

Some respondents noted having maintained mentorship interactions with former mentors (approx. 25% of respondents in quantitative questions did not, electronic supplementary material, figure S6). Some noted that they had a supportive graduate advisor and, while their formal obligations to each other ended with the mentee's graduation, mentor support and mentorship did not end. Some noted that despite their current mentorship situation being unhelpful, they still valued and received mentorship via email from their doctoral supervisor, some going on for decades. Reciprocally, some respondents noted having far more positive and pleasant interactions with their current faculty mentors despite unpleasant mentoring interactions during their training. Responders noted they valued mentors who prioritized mentee well-being over productivity. Responders also noted that they learned how to choose a mentor based on prior extremely negative mentor experiences. Some respondents mentioned mentors they had since graduate training that had guided the mentee through many aspects of their career development and job transitions.

### (l) Mid-career faculty also need mentors

The qualitative responses highlighted mid-career faculty as an overlooked community in mentoring relationships. Some tenured or established faculty noted that their mentor was their department chair, who was not a mentor in an official capacity, rather the only person they received substantial advice from since arrival. Some noted that in their institution, junior faculty had mentorship committees but that this was not the case for tenured faculty who joined the department. Some respondents noted that they mentor graduate students, postdoctoral researchers and tenure-track faculty, informally and as part of a mentoring programme, but that they had never themselves been offered any formal mentoring from their institution. Some noted benefiting from supportive professors externally who had been encouraging, but internally it seemed to be expected that faculty would not benefit from mentorship once they were promoted to a tenured or associate professor position, even though their funding opportunities are reduced compared with junior colleagues. Many regard mentorship for early (pre-tenure) faculty as much more available than mentorship for mid-career faculty. It is harder to find peer mentors within the institution to help guide the jump from associate to full professorship, and there are fewer online resources and tutorials on this transition. Thus, there is a need to facilitate continued mentorship for faculty members, even upon the transition to tenure.

## 4. Discussion

Our goal in conducting this study was to document the attributes and showcase the impact of faculty mentorship. We believe that illuminating the current state of faculty mentorship will draw attention to areas that require improvements to elevate faculty mentoring worldwide. The findings of this survey reveal interesting insights about the current state of faculty mentorship.

Beyond geographical differences, we found differences in how men and women faculty perceive mentorship. Female faculty indicated less satisfaction regarding the mentorship that they received and lower optimism on future career prospects than their male counterparts (electronic supplementary material, figures S4, S5, S14). This is consistent with differences previously observed in women faculties' perception of their work environment from their male counterparts [31] and additional obstacles women face in moving up the tenure ladder, in part due to work–family balance [32] and the academic workplace climate [33]. Survey respondents emphasized that having a diverse set of mentors that span a range of areas of expertise worked well to accommodate their needs. This often included multiple mentors—both formal and informal—to provide many potential sources for advice on different aspects of a scientist's job, as consistent with previous studies. For instance, junior faculty would almost certainly benefit from a mentor within their department to assist in navigating institutional guidelines and politics, but would additionally benefit from a senior mentor within their field to identify opportunities for networking, exposure, and promoting their research programme. Additionally, junior faculty would also likely benefit from a cohort of informal peer mentors that share resources and offer mutual learning to assist with questions on laboratory management, grant writing, and managing work–life balance. It would be highly improbable to find all of these qualities in just one person, highlighting the strengths of having a mentorship team to navigate being an academic faculty member. Indeed, our respondents noted that mentors are vital for success, and a number of respondents mentioned that without support from mentors they would have left academia. There are specific actions that can be taken at the departmental and institutional levels to effectively promote faculty mentorship. Departments should promote continuous and dynamic conversations on mentorship among colleagues and university administration, rather than promoting a ‘one size fits all’ mentorship solution. Along with discussing the merits of faculty membership, departments and institutions should provide protected time and training for mentors and mentees [34]. It is also important for departments and institutions to provide quality mentoring relationships, as described by our qualitative survey responses, as opposed to merely assigning faculty mentor(s) in the department. Departments should consult junior faculty on their needs and solicit their opinion (or, minimally, their approval) of proposed mentors. As there will be variations in mentee needs and mentor skills and knowledge, care and consideration should be taken to match junior faculty with senior faculty that can promote a positive mentoring experience.

### (a) Study strengths and limitations

In this work, we reported the findings of a large and independent survey of faculty members who may represent as many as 457 academic departments worldwide. While some departments and institutions may run faculty mentorship surveys internally, their findings are often not made public, suggesting a need for data curation on the broad needs of faculty mentorship. As our survey was completed before the COVID-19 pandemic, mentoring perceptions may have changed in ways that are not reflected in our survey. The COVID-19 pandemic also may have reduced mentoring, at least for those who were funded to travel to conferences and informally seek external mentorship. Our reach for faculty may have been influenced by our (the authors') life/biomedical sciences background, as our survey responses are highly enriched in faculty in the life and biomedical sciences and medical research currently residing in North America or Europe. The survey was presented in English, thus influencing our reach. Nations with English-speaking researchers, on social media and English-speaking universities, have the highest number of researchers per million inhabitants, so it is common for similar research culture surveys to have responses concentrated in North America and Europe. Despite this commonality, this concentration of responses influences the inferences we can make about mentorship relationships worldwide, particularly in geographical regions not well represented in our survey responses. Future surveys could focus on faculty mentorship experiences in specific countries and specific STEMM and non-STEMM disciplines. We did not collect data on the race or ethnicity of respondents and therefore cannot know how this may have influenced the findings of our survey. We also did not ask for specific information about the types of institutions that were surveyed, suggesting there may be undocumented differences between mentorship relationships at primarily research institutions (i.e., R1 institutions) versus primarily undergraduate institutions (i.e., R2 institutions). As the funding and tenure criteria are different at R1 and R2 institutions, this almost certainly affects mentorship needs of faculty, and future surveys could explore this aspect. Future surveys could also focus on faculty mentors, their mentorship training and their challenges and triumphs in the faculty-to-faculty mentoring interactions. Future research could also study how the mentorship challenges identified in this study may impact faculty mentee productivity, tenure and promotion outcomes and the mentorship of their mentees.

## Data Availability

The authors confirm that, for approved reasons, access restrictions apply to the data underlying the findings. Raw data underlying this study cannot be made publicly available in order to safeguard participant anonymity and that of their organizations. Ethical approval for the project was granted on the basis that only aggregated data are provided (with appropriate anonymization) as part of this publication. Supplementary material is available online [35].
